# Factors Facilitating and Hindering Development of a Medication Use Review Service in Eastern Europe and Iran-Cross-Sectional Exploratory Study

**DOI:** 10.3390/healthcare9091207

**Published:** 2021-09-14

**Authors:** Anita Tuula, Daisy Volmer, Liisa Jõhvik, Ieva Rutkovska, Indre Trečiokienė, Piotr Merks, Magdalena Waszyk-Nowaczyk, Mariola Drozd, Alena Tatarević, Maja Radovanlija, Carmen Pacadi, Arijana Meštrović, Réka Viola, Gyöngyvér Soós, Cristina Rais, Adriana-Elena Táerel, Magdalena Kuzelova, Marziyeh Zare, Payam Peymani, Marje Oona, Michael Scott

**Affiliations:** 1 Institute of Pharmacy, University of Tartu, 50411 Tartu, Estonia; daisy.volmer@ut.ee; 2 Hospital Pharmacy, Tartu University Hospital, 50406 Tartu, Estonia; liisa.johvik@kliinikum.ee; 3 Faculty of Pharmacy, Riga Stradins University, LV-1007 Riga, Latvia; ieva.rutkovska@gmail.com; 4 Pharmacy Center, Faculty of Medicine, Vilnius University, 01513 Vilnius, Lithuania; indre.treciokiene@mf.vu.lt; 5 Department of Pharmacology and Clinical Pharmacology, Faculty of Medicine, Collegium Medicum, Cardinal Stefan Wyszyński University in Warsaw, 01-938 Warsaw, Poland; p.merks@uksw.edu.pl; 6 Department of Pharmaceutical Technology, Pharmacy Practice Division, Poznan University of Medical Sciences, 60-780 Poznan, Poland; mwaszyk@ump.edu.pl; 7 Department of Humanities and Social Medicine, Medical University of Lublin, 20-093 Lublin, Poland; marioladrozd@umlub.pl; 8 Istrian Pharmacies, 52100 Pula, Croatia; tatarevic.app@gmail.com; 9 Pharmacy Rajić, 34000 Požega, Croatia; radovanlija.maja@gmail.com; 10 Mandis Pharm Community Pharmacies, 10000 Zagreb, Croatia; carmenpacadi@gmail.com; 11 Pharma Expert Consultancy and Education, 10040 Zagreb, Croatia; arijana.mestrovic@pharmaexpert.hr; 12 Faculty of Pharmacy, University of Szeged, H-6720 Szeged, Hungary; tothne.viola.reka@szte.hu (R.V.); SoosGyongyver@szte.hu (G.S.); 13 Faculty of Pharmacy, Carol Davila University of Medicine and Pharmacy, 020956 Bucharest, Romania; cristina_rais@yahoo.com (C.R.); adriana.taerel@yahoo.com (A.-E.T.); 14 Department of Pharmacology and Toxicology, Faculty of Pharmacy, Comenius University in Bratislava, 83232 Bratislava, Slovakia; kuzelova@fpharm.uniba.sk; 15 Health Policy Research Center, Institute of Health, Shiraz University of Medical Sciences, Shiraz 7134845794, Iran; marziyeh.zare70@gmail.com; 16 Rady Faculty of Health Sciences, College of Pharmacy, University of Manitoba, Winnipeg, MB R3E 0T5, Canada; peymani.payam@gmail.com; 17 Institute of Family Medicine and Public Health, University of Tartu, 50411 Tartu, Estonia; marje.oona@ut.ee; 18 Medicines Optimisation Innovation Centre, Antrim BT41 2RL, UK; drmichael.scott@northerntrust.hscni.net

**Keywords:** MUR, medication review, barriers, pharmacist, community pharmacy

## Abstract

Polypharmacy is a common issue in patients with chronic diseases. Eastern-European countries and Iran are exploring possibilities for implementing the Medication Use Review (MUR) as a measure for optimizing medication use and ensuring medication safety in polypharmacy patients. The aim of this study was to gain insights into the development of the community pharmacy sector and map facilitators and barriers of MUR in Eastern Europe and Iran. The representatives of the framework countries received a questionnaire on community pharmacy sector indicators, current and future developments of pharmacies, and factors encouraging and hindering MUR. To answer the questionnaire, all representatives performed document analysis, literature review, and qualitative interviews with key stakeholders. The socio-ecological model was used for inductive thematic analysis of the identified factors. Current community pharmacist competencies in framework countries were more related to traditional pharmacy services. Main facilitators of MUR were increase in polypharmacy and pharmaceutical waste, and access to patients’ electronic list of medications by pharmacists. Main barriers included the service being unfamiliar, lack of funding and private consultation areas. Pharmacists in the framework countries are well-placed to provide MUR, however, the service needs more introduction and barriers mostly on organizational and public policy levels must be addressed.

## 1. Introduction

In older patients with multiple chronic diseases, polypharmacy and drug-related problems are increasingly serious concerns. Polypharmacy patients have an increased risk of experiencing adverse drug reactions, geriatric syndromes, morbidity, and decreased medication adherence. Polypharmacy patients also receive inappropriate medications more frequently [1]. Although polypharmacy is often necessary, it is important to differentiate inappropriate polypharmacy, which occurs when the patient is receiving medication without an evidence-based indication, their medicines fail to achieve therapeutic goals, they experience or have a high risk of experiencing adverse drug reactions or they are not able to properly take their medications [2].

To support appropriate polypharmacy and ensure medication safety, many pharmacist-led services have been developed worldwide. Pharmacist-led medication review (MR) is a structured evaluation of a patient’s medicines for detecting drug-related problems and recommending interventions with the aim of optimizing medicine use and improving health outcomes [3]. MR services have been shown to improve medication awareness including improved medication adherence and decreased drug-related problems [4,5,6]. The latter is particularly important, as adverse drug events are the 14th leading cause of patient morbidity and mortality globally with a substantial proportion of such medication-related harm being avoidable [2]. Although the effect of MR on mortality, hospitalizations, length of stay in hospital, emergency department visits, readmissions, physician visits, and healthcare utilization needs more evidence [4,7,8], the World Health Organization in their 2019 report has considered it to be one of the key steps for assuring medication safety in polypharmacy [2].

Different types of MR services such as medication therapy management, home medicines review and medicines use review have been offered by community pharmacists in United States of America, Australia, New Zealand, and United Kingdom for many years [9,10,11,12]. By 2017, 19 of 34 European countries were offering a MR service and it has been recognized as one of the most commonly provided advanced pharmacist-led cognitive service in Europe [13]. Eastern European countries have historically been more focused on traditional services as dispensing, compounding, and counselling regarding medication use. Existing extended services mainly include point-of-care tests (e.g., taking blood pressure) and are focused on complementing the traditional pharmacy service rather than expanding pharmacists’ role in the broader healthcare system [14]. However, there have been some earlier attempts at applying an MR service in Bulgaria, Croatia, Czech Republic and Hungary [14,15].

One of the first Eastern-European countries to provide a nationally approved and funded MR service in community pharmacies was Slovenia in 2015 [13,16]. In relation to this service barriers to its provision were identified as lack of time in the pharmacy setting and recognition of the service by patients, physicians and health care payers. Positive patient feedback and extension of professional role were recognized as MR facilitators [16]. The main barriers to implementing MR identified in other studies have mostly been connected to pharmacy workflow, staffing issues, lack of access to patient’s clinical information and cooperation with other healthcare specialists [9,17,18].

## 2. Aim

The aim of this study was to map and analyze healthcare and pharmacy sector indicators facilitating and hindering provision of the Medication Use Review (MUR) service in Eastern-European countries and Iran.

## 3. Materials and Methods

Background information: In September 2017, a working group of pharmacists, general practitioners, and key stakeholders from both healthcare and the pharmacy sector was established in Estonia with the aim of developing a standard for the MUR service and identify possibilities for the implementation of the above-mentioned service. In 2018 the Estonian MR standard was adapted and amended from the 2013 Pharmaceutical Care Network Europe statement for medication review [19]. The adapted standard includes three levels of MR:

1. Simple MUR conducted in a community pharmacy by a community pharmacist; the pharmacist receives information about the medication regimen and patient’s diseases from the patient in a face-to-face interview and their general practitioner (GP); the service focuses on educating the patient on their diseases and medicines and detecting issues related to medication adherence and manifested drug related problems.
2. Comprehensive MR conducted in a community pharmacy by a community pharmacist who has passed an additional course in clinical pharmacy; additionally, requires information on the clinical test results from the GP as the pharmacist also evaluates the medication list for potential drug related problems.
3. MR service provided by a clinical pharmacist in the hospital setting; the clinical pharmacist additionally gets involved in establishing the treatment regimen for the patient [20].

In January 2019, the MUR pilot project started in Estonia and in March the international MUR network consisting of 11 countries (Estonia, Latvia, Lithuania, Poland, Croatia, Bosnia and Herzegovina, Hungary, Romania, Bulgaria, Slovakia and Iran) was launched to look further at the opportunities for advancing the MUR service on the first level according to the adapted standard, which can also be considered Type 2 MR according to Hatah et al. 2014 meta-analysis [21].

Study instrument: In September 2019, representatives of all 11 MUR framework countries received a study instrument compiled by researchers and practicing pharmacists from Estonia and consisting of the following questions:

- Country indicators: total population, gross national income per capita, life expectancy at birth male/female, quality life years male/female, total expenditure on health as percentage of gross domestic product—GDP (%), pharmaceutical spending as a percentage of health spending (%); share of population aged 65 and over (%), long term illness in elderly population (%).
- Pharmacy sector indicators: number of community pharmacies; number of community pharmacists; number of assistant pharmacists at community pharmacies.
- Current and future competencies and roles of community pharmacists; recent and future developments in community pharmacies.
- Factors which are facilitators and barriers to MUR.

To complete the questionnaire, the representatives of the MUR network were asked to use existing information and data sources specifically: literature review, document analysis and qualitative interviewing of key stakeholders (representatives of governmental institutions, professional organizations and higher education institutions providing pharmacy education). Interviews were conducted to answer the third and fourth question and were recorded by written notes. In May 2021, all representatives were asked for follow-up details on recent developments in the pharmacy sector and pharmacist’s role. Ethics committee approval was not sought for this type of research, as the data collected for the study are not sensitive personal information, nor were any interventions conducted on the study participants. All participants were informed their answers will be used in the study and their anonymity will be guaranteed prior to the interviews.

Data analysis: the parameters of pharmacy sector and country indicators for Iran were different from the Eastern-European framework countries and thus were not included in equations for expenditure on health, share of elderly population and community pharmacy sector indicators.

The social ecological model (SEM) including individual, interpersonal, institutional/organizational, public policy, and social domains [22] was adapted and used for the inductive thematic analysis to identify which are the main factors currently affecting pharmacists in providing the service and on what level these factors need to be addressed. SEM analysis was used for all factors that were detected by at least two framework countries. The standards for reporting qualitative research (SRQR) were used for reporting the research results [23].

## 4. Results

Answers were collected in November 2019 from nine out of the eleven framework countries namely Croatia, Estonia, Latvia, Lithuania, Hungary, Poland, Iran, Slovakia and Romania.

### 4.1. Profile of Participant Countries

An overview of the country profiles is shown in Table 1. The average expenditure on health as a percentage of GDP in Eastern-Europe framework countries is 6.2%, being highest in Hungary (7.2%) and lowest in Poland (4.9%); Iran is slightly differing from others at 8.1% and has not been included in the equations. The share of elderly people in Eastern-European framework countries range between 15.5–23.5%, and roughly around 50–80% of them have at least one long-term illness (data of four network countries). The number of inhabitants per pharmacy ranges from 1822 to 4237 in Eastern-European countries, being on average 2880; Iran again differs with 7005 inhabitants per pharmacy. The average number of pharmacists per community pharmacy is 2.2 and the average number of assistant pharmacists per community pharmacy is 1.9 in Eastern-European framework countries.

### 4.2. Current Competencies of Community Pharmacists

Current competencies and roles of community pharmacists in all MUR framework countries include dispensing and counselling of prescription and over-the-counter medicines and compounding of extemporaneous medicines. Pharmacists also offer reporting or patient assistance in reporting adverse drug reactions, and patient education on disease prevention and maintenance of health in most framework countries. Usually, some point-of-care testing is provided (most common services named were blood pressure measurement, cholesterol, blood sugar and hemoglobin). In Lithuania, community pharmacists are offering an asthma management service and patient education on inhaler use techniques. In Croatia, pharmacists are improving patients’ medication adherence by counselling service and sorting patients’ medicines into weekly dispensers, and recently started dispensing biological therapies and counseling patients regarding their safe and proper use.

### 4.3. Future Competencies

Most often named future competencies of community pharmacists in framework countries were provision of extended services such as MR, influenza vaccination, diabetes screening, smoking cessation, international normalized ratio (INR) measurements, and new medicines service. Additionally, in Poland, performing simple diagnostic tests such as blood pressure monitoring, cholesterol and glucose measurements is expected to be a future competency.

### 4.4. Recent Developments and Future Plans

The primary recent development in the pharmacy sector for Estonia and Hungary was the ownership reform of community pharmacies and prohibition of vertical integration between wholesale and retail sale of medicines. Recent development for Romania includes an online pharmacy which dispenses over-the-counter medicines and parapharmaceuticals. For Poland, the recent development would be introduction of an E-Prescribing system.

Both Croatia and Estonia have been actively participating in the development of the electronic cross-border health services, which allows continuity of care for EU citizens while travelling abroad in EU. By 2021, both countries have implemented the electronic cross-border e-prescription system and are one of the first countries to do so.

Croatian working group also named several new regulations in pharmacy policies as a future development and by 2021, Croatian government has proposed the National Recovery and Resilience Plan for 2021–2026 which includes monitoring the outcomes of outpatient treatment and controlling and preventing medicine shortages in community pharmacies.

Future plans for Iran include an E-Prescribing system reaching everywhere in the country. In Lithuania, it was planned to introduce a state-run pharmacy network where state-owned hospital pharmacies would have outpatient departments to fulfil community pharmacy duties.

### 4.5. Factors Facilitating and Hindering MUR Development

In the project countries, the most often cited factors that were facilitating MUR were increase in polypharmacotherapy and pharmaceutical waste and access to an electronic list of medicines and medical records by pharmacists. The most often reported barriers were the service being unfamiliar to both physicians and pharmacists, the financing model of MUR, high workload in pharmacies and lack of private consultation rooms for MUR service in some community pharmacies. The SEM analysis detects which factors are currently facilitating or hindering the implementation of MUR in the practice of a community pharmacist. For the SEM analysis of facilitators and barriers, see Table 2.

## 5. Discussion

The development of the pharmacy sector in Eastern Europe and Iran could be considered similar. Pharmacist competencies mostly include providing traditional services such as dispensing, consulting and compounding, and there has been little development in pharmacist role regarding extended services so far. Thus, the barriers and facilitators of MUR service for the community pharmacist in these countries can be described jointly.

In framework countries, the initiative to introduce MUR has come from academics and active pharmacists, who understand that both polypharmacotherapy and pharmaceutical waste are serious concerns in the region, which have not been properly addressed by government institutions [24]. For comparison, the initiative to start providing Medicines Use Review in the United Kingdom as a nationally funded extended pharmacy service came from the National Health Service in 2005. The service was applied as a national approach to reduce health care costs, improve patients’ management of their medicines and to introduced patient-centered services in community pharmacies [25,26]. The SEM analysis indicates that one of the most important barriers to the MUR service in Eastern Europe and Iran is the lack of support from policy makers. Key factors such as financing the service, creating a central system for documenting pharmacists’ interventions, and allowing access to patient data necessary for providing MUR can only be solved on a national level.

Funding the service could prove to be difficult to overcome, as the average expenditure on healthcare in Eastern Europe and Iran is lower than the European Union average of 9.9% in all framework countries [27]. This might indicate that governments are less likely to fund healthcare as a sector and thus not support the provision of national remuneration for MUR. Currently there was no funding for the pilot project in Eastern Europe and Iran, which was considered one of the main barriers to the work by eight out of nine framework countries. The provision of MUR takes time and effort; hence the service could not be offered routinely without remuneration.

Access to digital records of the patient’s medications is vital in order to be able to offer the MUR service and has been cited as a prime encouraging factor in previously published literature [28]. In some framework countries, these data are not available to the pharmacist. Although MUR could be provided by only accessing data that the GP and the patient have decided to share, it is not an ideal solution for providing the service long term, as the initiative for service must always come from the GP. A central documentation system for pharmacists’ interventions would support the service, as effectively communicating MUR results to the patient’s GP is important for achieving the best health outcomes.

The COVID-19 pandemic might have encouraged implementing pharmacist-led extended services on the public policy level in some countries. As an example, Polish community pharmacists can now independently prescribe medicines to themselves and their own family members [29].

One of the main barriers to MUR at the organizational level is the high workload of pharmacists. This issue could be solved by hiring more pharmacists, although the structure of the pharmacy sector in framework countries does not support this solution. In most framework countries, the number of patients per community pharmacy is rather low, while there are few pharmacists per community pharmacies. The large number of community pharmacies might hinder the development of MUR considering that it would therefore be difficult to find the additional workforce necessary for the extended services.

Other important organizational factors include lack of private rooms and electronical resources such as computers in community pharmacies. Community pharmacy owners might be more willing to invest in solving these organizational problems if the service received national remuneration. However, previous research has shown that lack of pharmacy staff, prioritization of other clinical activities and dissatisfaction with the consultation area seem to be persistent issues even in countries where MR has been funded [9,17,30].

Pharmacy chains have been known to promote implementing extended services such as MUR, especially with national funding. Promotion of MUR by pharmacy chains, however, contributes to the quality of the service being more questionable in the eyes of other healthcare professionals [9]. Some framework countries have recently applied restrictions to pharmacy ownership or are planning to establish a state-run pharmacy network. These changes could influence determining the national implementation but also the status of the service in the future.

Insufficient collaboration between general practitioners and pharmacists was reported as a barrier by only two framework countries. For the pilot in Eastern Europe and Iran, motivated and supportive physicians were included early on, which might have caused the misconception that opposition to the service by general practitioners would not be an issue. In previously published literature, lack of collaboration between healthcare professionals is often highlighted as one of the main barriers [9,17,30]. To address this potential issue, interprofessional education and collaborative practice between different healthcare workers, namely physicians, pharmacists, and nurses, could be developed much further in the framework countries, as earlier experience of working together can also support MR services [31].

MUR is still unfamiliar to many pharmacists and other health professionals, who could direct their patients to receive the service. More pharmacists need to provide MUR in order to normalize the service in the health sector. It is important for health workers to understand the potential benefits and have a positive experience with MUR to refer their patients to the service routinely. Thorough introduction is necessary for effective implementation of MUR in the framework countries.

According to the international vision and policy, the provision of professional services should be a priority for pharmacies and health systems. As with other health innovations, the implementation of professional pharmacy services is complex and represents an area in which pharmacy in the community has had limited experience [32]. Skills in areas such as leadership, task delegation, goal setting and teamwork seem equally important to pharmacists’ clinical skills when it comes to integrating a new service into everyday practice. IT tools for data collection, legal support, training, and education, are just some of the drivers (or barriers) to change [33].

## 6. Conclusions

Key stakeholders in Eastern Europe and Iran are exploring the possibilities to apply extended pharmacy services such as MUR into practice. As an increase in polypharmacotherapy and pharmaceutical waste are increasing concerns in MUR framework countries, it is important to routinely assess patients’ medication use. More health professionals need to be introduced to MUR and interprofessional practice which supports pharmacists working together with GPs. Pharmacists are in many ways well placed to provide MUR; however, it is necessary to gain government support and financing for the service in Eastern Europe and Iran. Several organizational barriers such as high workload and lack of private consultation rooms, as well of the lack of standards for the service need to be addressed for continuing with MUR.

## Figures and Tables

**Table 1:** Profile of MUR framework countries.

	Croatia	Estonia	Hungary	Iran	Latvia	Lithuania	Poland	Romania	Slovakia
Population	4,130,000	1,326,000	9,685,000	82,914,000	1,908,000	2,760,000	37,888,000	19,365,000	5,457,000
GNI per capita (PPP $)	30,680	39,070	34,020	12,950	32,540	38,530	33,770	32,850	32,920
Life expectancy in years (m/f)	75/81	74/83	72/79	N/A	70/80	71/81	74/82	72/79	74/81
Quality life years (m/f)	N/A	54/59	59/60	N/A	50.6/52.2	56/60	N/A	59/59	56.4/57.0
Total expenditure on health, % of GDP	6.8	6.7	7.2	8.1	5.9	6.4	4.9	5	6.7
Pharmaceutical spending, % of health spending	23.3	18.2	22	N/A	27.4	29.1	N/A	20–22	26.4
Share of population aged over 65 (%)	19.6	19.4	23.5	6.1	20.0	19.6	18.2	19	15.5
Long term illness in elderly (%)	N/A	81.5 *	N/A	N/A	60 **	53.2 *	N/A	N/A	69.7 *
Number of community pharmacies	1181	495	2286	11,836	776	1515	12,286	9300	1716
Number of community pharmacists	2884 ***	894	5571	19,680	1591	2721	26,022	22,500	4183
Number of assistant pharmacists	2872 ****	774	7200	-	1284	1900	33,297	10,000	2304

* Data on patients aged 65 and above. ** Data on patients aged 60 and above. *** As number of MPharm working in health care system. **** As number of pharmacists technicians working in health care system. GNI—gross national income, PPP—purchasing power parity; m/f—male/female, N/A—not available.

**Table 2:** SEM for factors facilitating and hindering MUR service and the number of framework countries who detected the factor.

Domain	Barriers	Facilitators
First level individual	MUR service is not familiar to pharmacists (9) Lack of motivation in pharmacists (2)	Increased understanding about the importance and effectiveness of MUR service among pharmacists (3) Improved knowledge of pharmacists and physicians about the newest guidelines for pharmacotherapy and MUR service (2)
Second level intrapersonal	MUR service is not familiar to physicians (9) Insufficient collaboration between GPs and pharmacists (2)	Enhanced collaboration between healthcare professionals (3) Increased understanding about the importance and effectiveness of MUR service among physicians (3) Enhanced relations between pharmacists and patients (2) Development of the pharmacist role in the healthcare team (2)
Third level organizational/ institutional	Lack of private rooms and electronic resources in community pharmacies (7) Service standard needs further development (7) High workload of pharmacists (6)	Developed MUR service tool (3)
Fourth level public policy	Financing model for the MUR service (8) Limited access to patient data for pharmacists (3) Pharmacists have no central system for documentation of patient data and pharmacist interventions (3)	Access to an electronic list of medicines and medical records or electronic prescriptions by pharmacists (5)
Fifth level society	MUR is unfamiliar to patients (3)	Increase in polypharmacotherapy and pharmaceutical waste (8) Increase in population ageing and number of patients with chronic illness (2) The results of MUR can support the development of new aspects in pharmacotherapy such as personalized medicines (2) MUR program can increase the adherence of patients to therapies as they can better understand the disease and medication (2)

## Data Availability

Data is contained within the article.
